# Diaqua­(6-bromo­picolinato-κ^2^ *N*,*O*)(nitrato-κ^2^ *O*,*O*)copper(II)

**DOI:** 10.1107/S160053681100064X

**Published:** 2011-01-12

**Authors:** Joana A. Silva, Ana Pereira Magalhães, Manuela Ramos Silva, Abílio J. F. N. Sobral, Laura C. J. Pereira

**Affiliations:** a Chemistry Department, Faculdade de Ciências e Tecnologia, Universidade de Coimbra, P-3004-535 Coimbra, Portugal; b CEMDRX, Physics Department, University of Coimbra, P-3004-516 Coimbra, Portugal; c Instituto Tecnologico e Nuclear, Estrada Nacional 10, P-2686-953 Sacavem, Portugal

## Abstract

In the monomeric title complex, [Cu(C_6_H_3_BrNO_2_)(NO_3_)(H_2_O)_2_], the Cu^II^ ion is coordinated by a bidentate 6-bromo­picolinate ion, one nitrate ion and two water mol­ecules in a geometry inter­mediate between five- and six-coordinate. Conventional O—H⋯O hydrogen bonds link the complex mol­ecules, forming layers parallel to the *ab* plane.

## Related literature

For general background to copper complexes with low-dimensionality synthesized by our group, see: Martins, Ramos Silva *et al.* (2008 ▶), Martins, Silva *et al.* (2008 ▶); Ramos Silva *et al.* (2001*a* ▶,*b* ▶,*c* ▶, 2005*a* ▶,*b* ▶). For a magnetic low-dimensional system with picolinic acid, see: Eppley *et al.* (1997 ▶). For a similar compound with magnetic properties, see: Kukovec *et al.* (2008 ▶).
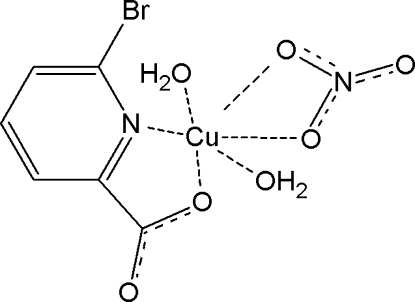


## Experimental

### 

#### Crystal data

[Cu(C_6_H_3_BrNO_2_)(NO_3_)(H_2_O)_2_]
*M* *_r_* = 362.59
Orthorhombic, 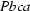

*a* = 9.0791 (14) Å
*b* = 14.035 (2) Å
*c* = 17.165 (2) Å
*V* = 2187.2 (6) Å^3^
*Z* = 8
Mo *K*α radiation
μ = 5.68 mm^−1^
*T* = 293 K
0.40 × 0.10 × 0.08 mm

#### Data collection

Bruker APEX CCD area-detector diffractometer
Absorption correction: multi-scan (*SADABS*; Sheldrick, 2000 ▶) *T*_min_ = 0.619, *T*_max_ = 0.999
35919 measured reflections
3263 independent reflections
1849 reflections with *I* > 2σ(*I*)
*R*_int_ = 0.069

#### Refinement

*R*[*F* ^2^ > 2σ(*F* ^2^)] = 0.038
*wR*(*F* ^2^) = 0.137
*S* = 1.03
3263 reflections
167 parameters
7 restraints
H atoms treated by a mixture of independent and constrained refinement
Δρ_max_ = 1.06 e Å^−3^
Δρ_min_ = −1.19 e Å^−3^

### 

Data collection: *SMART* (Bruker, 2003 ▶); cell refinement: *SAINT* (Bruker, 2003 ▶); data reduction: *SAINT*; program(s) used to solve structure: *SHELXS97* (Sheldrick, 2008 ▶); program(s) used to refine structure: *SHELXL97* (Sheldrick, 2008 ▶); molecular graphics: *ORTEPII* (Johnson, 1976 ▶); software used to prepare material for publication: *SHELXL97*.

## Supplementary Material

Crystal structure: contains datablocks global, I. DOI: 10.1107/S160053681100064X/bt5450sup1.cif

Structure factors: contains datablocks I. DOI: 10.1107/S160053681100064X/bt5450Isup2.hkl

Additional supplementary materials:  crystallographic information; 3D view; checkCIF report

## Figures and Tables

**Table 1:** Hydrogen-bond geometry (Å, °)

*D*—H⋯*A*	*D*—H	H⋯*A*	*D*⋯*A*	*D*—H⋯*A*
O6—H6*A*⋯O3^i^	0.85 (1)	2.03 (2)	2.825 (4)	156 (4)
O6—H6*B*⋯O2^ii^	0.85 (2)	1.86 (1)	2.700 (4)	166 (2)
O7—H7*A*⋯O4^iii^	0.85 (1)	2.06 (2)	2.874 (5)	163 (5)
O7—H7*B*⋯O1^ii^	0.85 (3)	2.08 (3)	2.833 (4)	148 (5)

Symmetry codes: (i) 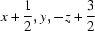; (ii) 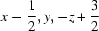; (iii) 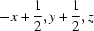.

**Fig. 1.**
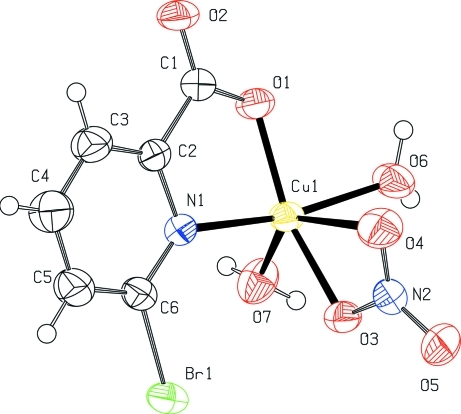
ORTEPII (Johnson, 1976) plot of the title compound. Displacement ellipsoids are drawn at the 50% level.

**Fig. 2.**
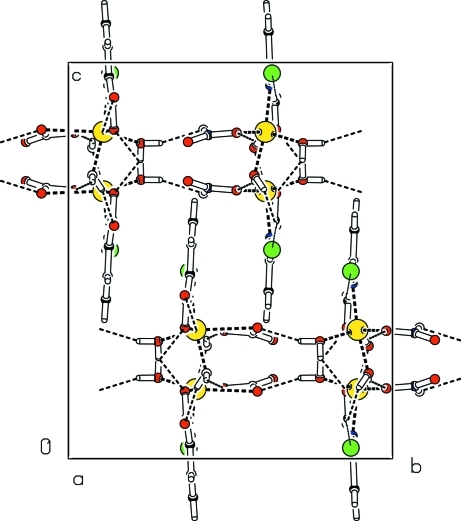
Packing of the molecules in the unit cell showing the H-bonds as dashed lines.

## supplementary crystallographic information

### Comment

The title compound was obtained within a project of synthesizing new molecular magnets (Martins, Silva *et al.*, 2008; Martins, Ramos Silva *et al.*, 2008; Ramos Silva *et al.*, 2001*a*, 2001*b*, 2001*c*, 2005*a*, 2005*b*). Molecular based magnets can capitalize on the flexibility inherent in carbon chemistry. Such flexibility allows a rational choice of ligands to control the dimensionality of the system, so that quantum effects can be enhanced. Picolinic and hydroxypicolinic acid have been widely used as ligands in low- dimensional metallic systems (Eppley *et al.*, 1997) but 6-bromopicolinic acid has been scarcely used. A different substituent in the pyridine ring may lead to significant electronic and steric effects enlarging the structural diversity. In fact, Kukovec *et al.* (2008) synthesized a copper (II) complex with 6-bromopicolinic acid as a bidentate ligand in which the magnetic exchange pathway is connected to the Br···π interaction.

In the title compound, the Cu^II^ ion is coordinated by a bromopicolinate ligand, two water molecules and a nitrate ion (Fig. 1). One of the Cu—O bonds is rather long [Cu1—O4 2.682 (3) °] so that the coordination about the copper ion is intermediate between five and six-coordination. If the latter bond is to be ignored, the remaining coordination stereochemistry is near a square pyramid. In that case, the copper ion is 0.3149 (5) Å above the least-squares plane of the basal coordinating atoms. The H-bond network is confined to layers parallel to the *ab* plane (Fig. 2, Table 1). The bromine also forms a short contact [3.066 (2) Å] with O2^i^ [symmetry code: (i) -1 + *x*,*y*,*z*].

The magnetic susceptibility was measured using a SQUID magnetometer in function of temperature with an applied magnetic field of 2 T. The inverse susceptibility showed a linear dependence with temperature, excluding any interaction between magnetic centers.

### Experimental

0.14 mmol of 6-bromo-2-pyridinecarboxaldehyde in dichloromethane (10 ml) was added to 0.12 mmol of Cu(NO3)2.3H2O in water (10 ml). After a few weeks, blue single crystals of the title compound were obtained.

### Refinement

H atoms bound to C atoms were placed at calculated positions and were treated as riding on the parent atoms with C—H = 0.93 Å (aromatic) and with *U*_iso_(H) = 1.2 *U*_eq_(C). H atoms of water molecules O6 an O7 could not be correctly located in a difference Fourier map. They were placed at positions calculated to optimize H-bonds and refined using  restraints [O—H = 0.85 (1) Å, H—H = 1.34 (1) Å] and *U*_iso_(H) = 1.5Ueq(O). To avoid that water (O6) H atoms slip into density peaks around the heavy metal atom, a *DFIX* command was used to garantee a Cu···H distance of at least 2.50 (1) Å. There are maximum and minimum density peaks slightly above 1 e/Å^3^.

### Crystal data

**Table:**

[Cu(C_6_H_3_BrNO_2_)(NO_3_)(H_2_O)_2_]	*F*(000) = 1416
*M**_r_* = 362.59	*D*_x_ = 2.202 Mg m^−^^3^
Orthorhombic, *P**b**c**a*	Mo *K*α radiation, λ = 0.71073 Å
Hall symbol: -P 2ac 2ab	Cell parameters from 6959 reflections
*a* = 9.0791 (14) Å	θ = 2.9–26.3°
*b* = 14.035 (2) Å	µ = 5.68 mm^−^^1^
*c* = 17.165 (2) Å	*T* = 293 K
*V* = 2187.2 (6) Å^3^	Needle, blue
*Z* = 8	0.40 × 0.10 × 0.08 mm

### Data collection

**Table:**

Bruker APEX CCD area-detector diffractometer	3263 independent reflections
Radiation source: fine-focus sealed tube	1849 reflections with *I* > 2σ(*I*)
graphite	*R*_int_ = 0.069
φ and ω scans	θ_max_ = 31.4°, θ_min_ = 2.4°
Absorption correction: multi-scan (*SADABS*; Sheldrick, 2000)	*h* = −12→11
*T*_min_ = 0.619, *T*_max_ = 0.999	*k* = −19→20
35919 measured reflections	*l* = −24→24

### Refinement

**Table:**

Refinement on *F*^2^	Secondary atom site location: difference Fourier map
Least-squares matrix: full	Hydrogen site location: inferred from neighbouring sites
*R*[*F*^2^ > 2σ(*F*^2^)] = 0.038	H atoms treated by a mixture of independent and constrained refinement
*wR*(*F*^2^) = 0.137	*w* = 1/[σ^2^(*F*_o_^2^) + (0.0722*P*)^2^] where *P* = (*F*_o_^2^ + 2*F*_c_^2^)/3
*S* = 1.03	(Δ/σ)_max_ = 0.001
3263 reflections	Δρ_max_ = 1.06 e Å^−^^3^
167 parameters	Δρ_min_ = −1.19 e Å^−^^3^
7 restraints	Extinction correction: *SHELXL97* (Sheldrick, 2008), Fc^*^=kFc[1+0.001xFc^2^λ^3^/sin(2θ)]^-1/4^
Primary atom site location: structure-invariant direct methods	Extinction coefficient: 0.0019 (3)

### Special details

**Table:**

Geometry. All e.s.d.'s (except the e.s.d. in the dihedral angle between two l.s. planes) are estimated using the full covariance matrix. The cell e.s.d.'s are taken into account individually in the estimation of e.s.d.'s in distances, angles and torsion angles; correlations between e.s.d.'s in cell parameters are only used when they are defined by crystal symmetry. An approximate (isotropic) treatment of cell e.s.d.'s is used for estimating e.s.d.'s involving l.s. planes.
Refinement. Refinement of *F*^2^ against ALL reflections. The weighted *R*-factor *wR* and goodness of fit *S* are based on *F*^2^, conventional *R*-factors *R* are based on *F*, with *F* set to zero for negative *F*^2^. The threshold expression of *F*^2^ > σ(*F*^2^) is used only for calculating *R*-factors(gt) *etc*. and is not relevant to the choice of reflections for refinement. *R*-factors based on *F*^2^ are statistically about twice as large as those based on *F*, and *R*- factors based on ALL data will be even larger.

### Fractional atomic coordinates and isotropic or equivalent isotropic displacement parameters (Å^2^)

**Table:**

	*x*	*y*	*z*	*U*_iso_*/*U*_eq_	
Cu1	0.27639 (5)	0.10769 (4)	0.67505 (3)	0.03377 (16)	
Br1	−0.01236 (4)	0.12831 (4)	0.52718 (3)	0.04471 (16)	
C1	0.5394 (5)	0.1362 (3)	0.6017 (2)	0.0356 (10)	
C2	0.4323 (4)	0.1249 (2)	0.5356 (2)	0.0291 (8)	
C3	0.4765 (5)	0.1207 (4)	0.4602 (3)	0.0463 (12)	
H3	0.5763	0.1214	0.4479	0.056*	
C4	0.3723 (6)	0.1155 (4)	0.4020 (3)	0.0542 (14)	
H4	0.4004	0.1107	0.3500	0.065*	
C5	0.2261 (5)	0.1175 (4)	0.4223 (3)	0.0500 (13)	
H5	0.1533	0.1164	0.3843	0.060*	
C6	0.1890 (5)	0.1212 (3)	0.5006 (2)	0.0367 (10)	
O1	0.4821 (3)	0.1405 (2)	0.66941 (16)	0.0406 (8)	
O2	0.6707 (3)	0.1410 (3)	0.58800 (18)	0.0529 (9)	
O3	0.1019 (3)	0.0189 (2)	0.67729 (14)	0.0378 (7)	
O4	0.2795 (3)	−0.0834 (3)	0.66960 (18)	0.0516 (9)	
O5	0.0612 (4)	−0.1309 (2)	0.7005 (2)	0.0572 (9)	
O6	0.3112 (3)	0.0737 (3)	0.78475 (15)	0.0505 (9)	
H6A	0.3992 (12)	0.074 (3)	0.8019 (8)	0.076*	
H6B	0.264 (3)	0.103 (2)	0.8203 (5)	0.076*	
O7	0.1350 (4)	0.2239 (2)	0.70395 (19)	0.0494 (8)	
H7A	0.156 (5)	0.2826 (10)	0.703 (3)	0.074*	
H7B	0.086 (4)	0.222 (4)	0.7459 (14)	0.074*	
N1	0.2882 (4)	0.1232 (2)	0.55771 (19)	0.0313 (8)	
N2	0.1477 (4)	−0.0683 (3)	0.68317 (17)	0.0356 (8)	

### Atomic displacement parameters (Å^2^)

**Table:**

	*U*^11^	*U*^22^	*U*^33^	*U*^12^	*U*^13^	*U*^23^
Cu1	0.0240 (3)	0.0461 (4)	0.0312 (2)	0.0008 (2)	0.00166 (17)	0.0011 (2)
Br1	0.0287 (2)	0.0573 (3)	0.0481 (3)	0.00161 (19)	−0.00589 (16)	0.0033 (2)
C1	0.030 (2)	0.040 (3)	0.037 (2)	−0.0019 (18)	−0.0022 (16)	0.0035 (17)
C2	0.028 (2)	0.022 (2)	0.0374 (19)	0.0017 (16)	0.0021 (15)	0.0012 (15)
C3	0.035 (3)	0.063 (3)	0.041 (2)	−0.002 (2)	0.0043 (17)	−0.001 (2)
C4	0.049 (3)	0.079 (4)	0.035 (2)	−0.004 (2)	0.0032 (19)	−0.007 (2)
C5	0.043 (3)	0.070 (4)	0.037 (2)	−0.004 (2)	−0.0053 (18)	0.002 (2)
C6	0.032 (2)	0.045 (3)	0.0328 (19)	0.0002 (17)	−0.0041 (16)	0.0018 (17)
O1	0.0272 (15)	0.064 (2)	0.0312 (14)	−0.0077 (14)	0.0003 (10)	−0.0009 (13)
O2	0.0238 (16)	0.091 (3)	0.0443 (17)	−0.0025 (15)	0.0015 (12)	0.0099 (16)
O3	0.0269 (14)	0.0397 (19)	0.0468 (15)	0.0008 (12)	−0.0003 (11)	0.0047 (12)
O4	0.0397 (19)	0.049 (2)	0.066 (2)	0.0137 (15)	0.0097 (14)	0.0014 (16)
O5	0.055 (2)	0.052 (2)	0.065 (2)	−0.0217 (17)	−0.0069 (17)	0.0160 (16)
O6	0.0313 (16)	0.087 (3)	0.0326 (14)	0.0088 (16)	0.0015 (11)	0.0027 (16)
O7	0.0525 (19)	0.0418 (19)	0.0541 (17)	0.0043 (16)	0.0184 (14)	−0.0001 (16)
N1	0.0277 (17)	0.032 (2)	0.0340 (16)	−0.0022 (14)	0.0002 (13)	0.0037 (13)
N2	0.037 (2)	0.036 (2)	0.0337 (16)	0.0017 (17)	−0.0030 (14)	0.0025 (14)

### Geometric parameters (Å, °)

**Table:**

Cu1—O1	1.926 (3)	C4—C5	1.373 (7)
Cu1—O6	1.968 (3)	C4—H4	0.9300
Cu1—O3	2.016 (3)	C5—C6	1.386 (6)
Cu1—N1	2.029 (3)	C5—H5	0.9300
Cu1—O7	2.134 (3)	C6—N1	1.332 (5)
Br1—C6	1.886 (4)	O3—N2	1.296 (4)
C1—O2	1.218 (5)	O4—N2	1.238 (4)
C1—O1	1.274 (5)	O5—N2	1.215 (5)
C1—C2	1.503 (6)	O6—H6A	0.851 (9)
C2—C3	1.356 (6)	O6—H6B	0.85 (2)
C2—N1	1.363 (5)	O7—H7A	0.845 (10)
C3—C4	1.378 (6)	O7—H7B	0.85 (3)
C3—H3	0.9300		
			
O1—Cu1—O6	87.13 (12)	C3—C4—H4	120.7
O1—Cu1—O3	155.59 (13)	C4—C5—C6	118.9 (4)
O6—Cu1—O3	87.61 (12)	C4—C5—H5	120.6
O1—Cu1—N1	82.72 (12)	C6—C5—H5	120.6
O6—Cu1—N1	165.35 (13)	N1—C6—C5	123.3 (4)
O3—Cu1—N1	97.29 (11)	N1—C6—Br1	118.4 (3)
O1—Cu1—O7	114.35 (14)	C5—C6—Br1	118.2 (3)
O6—Cu1—O7	93.40 (13)	C1—O1—Cu1	115.5 (3)
O3—Cu1—O7	89.74 (13)	N2—O3—Cu1	109.4 (2)
N1—Cu1—O7	100.38 (13)	Cu1—O6—H6A	118.7 (11)
O2—C1—O1	125.0 (4)	Cu1—O6—H6B	118.9 (11)
O2—C1—C2	119.6 (4)	H6A—O6—H6B	103.2 (14)
O1—C1—C2	115.5 (4)	Cu1—O7—H7A	127 (3)
C3—C2—N1	123.3 (4)	Cu1—O7—H7B	119 (3)
C3—C2—C1	122.3 (4)	H7A—O7—H7B	99 (4)
N1—C2—C1	114.3 (3)	C6—N1—C2	116.4 (3)
C2—C3—C4	119.4 (4)	C6—N1—Cu1	133.9 (3)
C2—C3—H3	120.3	C2—N1—Cu1	109.2 (2)
C4—C3—H3	120.3	O5—N2—O4	123.1 (4)
C5—C4—C3	118.6 (4)	O5—N2—O3	119.7 (4)
C5—C4—H4	120.7	O4—N2—O3	117.2 (3)
			
O2—C1—C2—C3	−0.6 (6)	O7—Cu1—O3—N2	−162.2 (2)
O1—C1—C2—C3	179.0 (4)	C5—C6—N1—C2	2.3 (6)
O2—C1—C2—N1	−178.1 (4)	Br1—C6—N1—C2	−175.4 (3)
O1—C1—C2—N1	1.5 (5)	C5—C6—N1—Cu1	−168.5 (3)
N1—C2—C3—C4	0.7 (7)	Br1—C6—N1—Cu1	13.8 (5)
C1—C2—C3—C4	−176.6 (4)	C3—C2—N1—C6	−2.8 (6)
C2—C3—C4—C5	1.9 (7)	C1—C2—N1—C6	174.7 (3)
C3—C4—C5—C6	−2.3 (7)	C3—C2—N1—Cu1	170.2 (3)
C4—C5—C6—N1	0.2 (7)	C1—C2—N1—Cu1	−12.3 (4)
C4—C5—C6—Br1	177.9 (4)	O1—Cu1—N1—C6	−174.5 (4)
O2—C1—O1—Cu1	−169.1 (4)	O6—Cu1—N1—C6	139.0 (5)
C2—C1—O1—Cu1	11.3 (5)	O3—Cu1—N1—C6	30.1 (4)
O6—Cu1—O1—C1	155.0 (3)	O7—Cu1—N1—C6	−60.9 (4)
O3—Cu1—O1—C1	77.1 (4)	O1—Cu1—N1—C2	14.3 (2)
N1—Cu1—O1—C1	−14.5 (3)	O6—Cu1—N1—C2	−32.2 (6)
O7—Cu1—O1—C1	−112.6 (3)	O3—Cu1—N1—C2	−141.1 (2)
O1—Cu1—O3—N2	8.9 (4)	O7—Cu1—N1—C2	127.8 (2)
O6—Cu1—O3—N2	−68.8 (2)	Cu1—O3—N2—O5	165.8 (3)
N1—Cu1—O3—N2	97.3 (2)	Cu1—O3—N2—O4	−15.0 (4)

### Hydrogen-bond geometry (Å, °)

**Table:**

*D*—H···*A*	*D*—H	H···*A*	*D*···*A*	*D*—H···*A*
O6—H6A···O3^i^	0.85 (1)	2.03 (2)	2.825 (4)	156 (4)
O6—H6B···O2^ii^	0.85 (2)	1.86 (1)	2.700 (4)	166 (2)
O7—H7A···O4^iii^	0.85 (1)	2.06 (2)	2.874 (5)	163 (5)
O7—H7B···O1^ii^	0.85 (3)	2.08 (3)	2.833 (4)	148 (5)

Symmetry codes: (i) *x*+1/2, *y*, −*z*+3/2; (ii) *x*−1/2, *y*, −*z*+3/2; (iii) −*x*+1/2, *y*+1/2, *z*.
